# Splenic Injury After a Screening Colonoscopy

**DOI:** 10.7759/cureus.31004

**Published:** 2022-11-02

**Authors:** Muhammad S Aleem, Sravan Ponnekanti, Bikal Lamichhane, Maheen Anwar, Subash Ghimire, Victor Kolade

**Affiliations:** 1 Medicine, Jinnah Hospital/Allama Iqbal Medical College, Lahore, PAK; 2 Internal Medicine, Guthrie Robert Packer Hospital, Sayre, USA

**Keywords:** splenic haematoma, screening colonoscopy, splenic trauma, screening, colonoscopy

## Abstract

Colonoscopy is a safe and routinely performed procedure worldwide. However, complications such as bleeding and perforation can occur. Splenic injury after a colonoscopy is a rare complication. We present the case of a 71-year-old woman who presented to the ED due to abdominal pain after undergoing a screening colonoscopy. An abdominal CT scan showed a grade III splenic injury with a subcapsular hematoma. She was successfully managed conservatively. Splenic injuries after colonoscopy are associated with significant morbidity and mortality. A low threshold of suspicion and timely diagnosis can improve outcomes.

## Introduction

Colonoscopy is a safe and routinely performed procedure for screening, diagnosing, and treating colonic disease. However, splenic injury after colonoscopy is a rare but potentially life-threatening complication. First reported in 1974, and its incidence has increased since then, with 73 cases reported so far [1-8]. Here, we describe a case of subcapsular splenic hematoma after a screening colonoscopy.

## Case presentation

**Figure 1:**
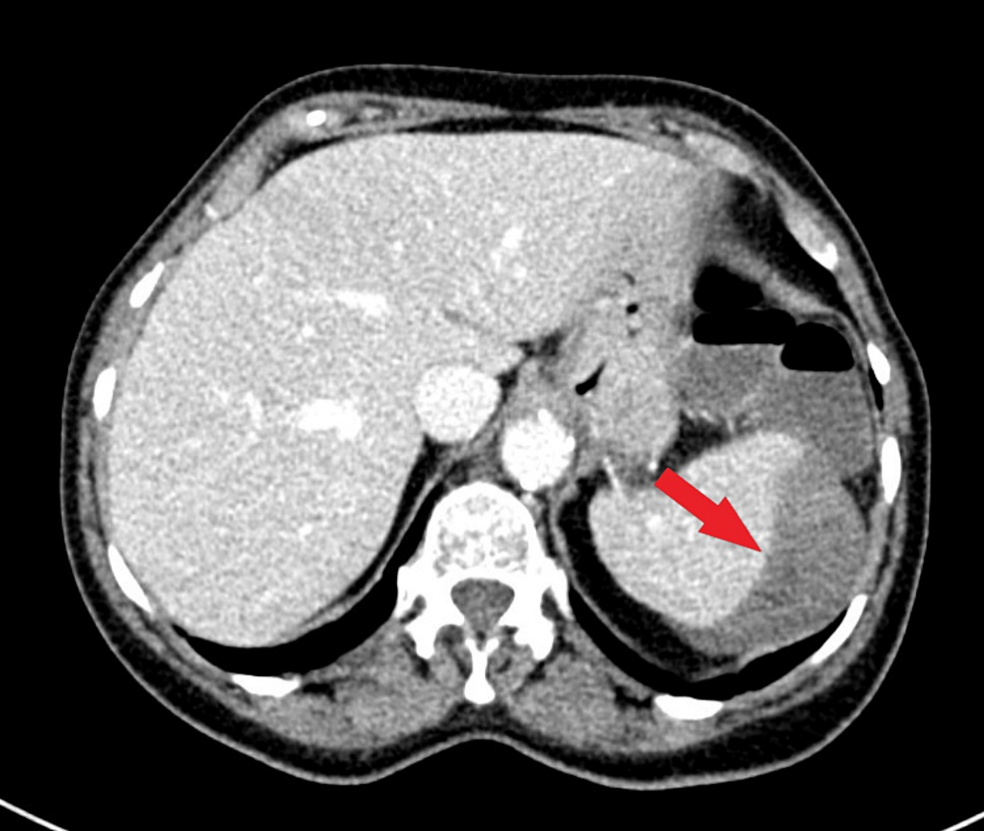
CT abdomen axial section showing subcapsular splenic hematoma (red arrow).

**Figure 2:**
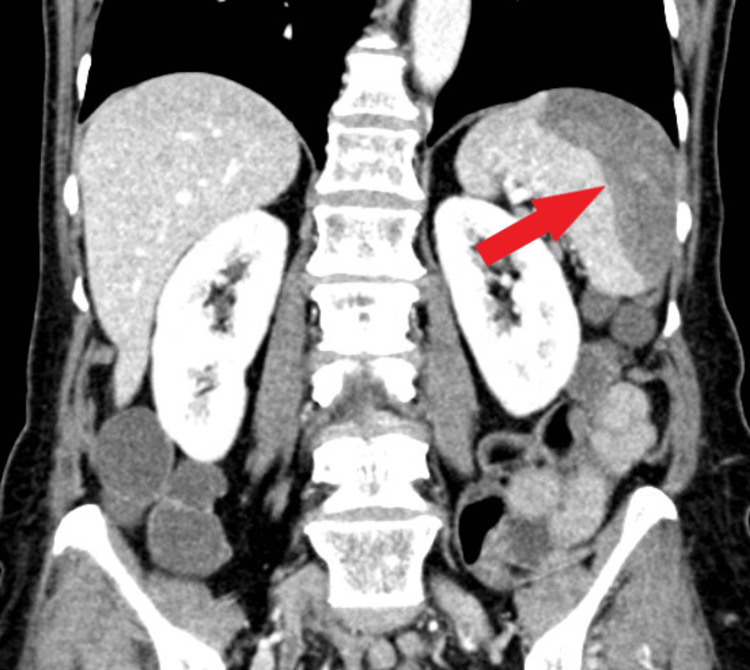
CT abdomen coronal section showing subcapsular splenic hematoma (red arrow).

A 71-year-old postmenopausal female with a past medical history of tobacco use, hyperlipidemia, varicose veins, appendectomy, inguinal hernia repair, caesarian section, and fallopian tube ligation presented with a complaint of worsening left upper quadrant abdominal pain. She had undergone a screening colonoscopy 10 hours prior, which was uneventful. The abdominal pain was insidious onset, non-radiating, unrelated to posture, and associated with nausea. Her vital signs were stable on presentation, including a blood pressure of 125/64 and a 79 beats per minute heart rate. Physical examination was significant for left upper and lower quadrant tenderness in the abdomen. Labs were significant for hemoglobin which trended from 13 to 11.4 within 12 hours. A CT scan of the abdomen and pelvis with IV contrast revealed a grade III splenic laceration in the form of a large 7.2 x 4.8 x 9.0 cm subcapsular splenic hematoma encompassing greater than 50% of the splenic surface without intraparenchymal contusion, laceration or involvement of the hilar vasculature, or pseudoaneurysm of a retroperitoneal hematoma (Figures 1-2). She was resuscitated with 0.9% IV saline. General surgery was consulted, and conservative management was recommended with a low threshold for surgical interventional radiology intervention if required. She was admitted for observation, and her hemoglobin was monitored every six hours. Her vitals and hemoglobin remained stable throughout her hospital stay. She was discharged in stable condition with advice to avoid strenuous physical activity for four weeks. At the two-month follow-up, she was asymptomatic except for mild postprandial epigastric fullness and seemed to be doing well.

## Discussion

A colonoscopy is a routine, low-risk procedure used to diagnose and treat colonic diseases [1, 2]. Since its inception in 1969, colonoscopies have been increasing. For example, screening colonoscopies increased by 21% from 2000 to 2013 [3, 4]. As colonoscopies performed each year increase, so does the number of complications. Bleeding is the most common complication after a colonoscopy, with an incidence rate of up to 6% of cases. Perforation is a relatively rare yet dreaded complication with an incidence of less than 1 in 500 of all cases (<0.2%) [1]. Splenic injury after a colonoscopy is an infrequent complication with an estimated incidence of 1 in 100000 patients (0.001%) [3]. Since the first reported case of splenic injury after a colonoscopy by Wherry DC and Zehner H in 1974 [5], 73 more cases have been reported in the literature [2, 3, 6-9]. The mechanism behind splenic injury after a colonoscopy is poorly understood. One possible reason is excessive traction on the splenocolic ligament or splenic adhesions secondary to previous abdominal surgery or inflammation. Direct, blunt trauma during the navigation of the colonoscope through the splenic flexure could also be a reason [3]. Other risk factors reported for splenic injury include older age, female sex, biopsies or polypectomy in the transverse colon, splenomegaly, underlying splenic disease, inexperienced endoscopist, or a technically demanding procedure [3, 5].

As per a systematic review by Ullah W et al., 46% of the colonoscopies leading to splenic injury were performed for screening, and 28% for evaluation of GI bleeding. However, no association between the indication for colonoscopy and the rate of splenic trauma was observed [3]. Other less common indications for colonoscopy included abdominal pain, weight loss, polypectomy, and constipation [3, 5]. More than two-thirds of the patients present with either generalized abdominal pain or left upper quadrant pain. Other less common presentations included dizziness, syncope, and back or chest pain [2, 3, 5-7]. One case reported left shoulder pain as the initial presentation two hours after a screening colonoscopy [8]. While most patients develop symptoms within 24 hours of colonoscopy, it can take up to 72 hours to develop symptoms after the procedure [2, 5]. Splenic rupture was seen in around 22% of the patients, with the remaining patients having various splenic injuries, including subcapsular hematomas, such as in our patient [3]. Up to 40% of the patients can be hemodynamically unstable on presentation [7]. Although its sensitivity can be limited by bowel dilatation after colonoscopy, USG should be performed in hemodynamically unstable patients. In hemodynamically stable patients, CT imaging is the imaging modality of choice [2, 5]. Management depends on the clinical presentation, hemodynamic stability, and type of splenic injury found in CT imaging. A total of 47% of patients (n=35/74) required a splenectomy, with the majority requiring a laparotomy. A total of 39% (n=29/74) of the patients, including ours, were managed conservatively with close monitoring and serial examinations, IV fluids, blood transfusions, and analgesics. Angioembolization by interventional radiology should also be considered in hemodynamically stable patients with active contrast extravasation on imaging [2, 3, 5-8]. The overall mortality rate from splenic injury after a colonoscopy is reported at around 10%. Delayed presentation and splenic rupture are associated with a higher mortality rate [3, 7].

## Conclusions

As the rate of colonoscopies increases, the rate of complications will increase. Splenic injury is an infrequent complication associated with significant morbidity and mortality. Timely diagnosis and management can lead to better outcomes. It should be suspected in any patient who presents with abdominal, shoulder, or chest pain or dizziness after a recent colonoscopy. Post-procedure review of complications as a quality improvement tool and educating the endoscopists will go a long way toward improving the overall quality of the endoscopy.
